# Androgen-targeted therapy in men with prostate cancer: evolving practice and future considerations

**DOI:** 10.1038/s41391-018-0079-0

**Published:** 2018-08-21

**Authors:** E. David Crawford, Axel Heidenreich, Nathan Lawrentschuk, Bertrand Tombal, Antonio C. L. Pompeo, Arturo Mendoza-Valdes, Kurt Miller, Frans M. J. Debruyne, Laurence Klotz

**Affiliations:** 1 University of Colorado School of Medicine, Denver, CO USA; 2 University of Cologne, Cologne, Germany; 3 Medical University of Vienna, Vienna, Austria; 4 University of Melbourne, Melbourne, Australia; 5 Cliniques Universitaires Saint-Luc, Brussels, Belgium; 6 Faculdade de Medicina do ABC, São Paulo, Brazil; 7 Hospital Médica Sur, Ciudad de México, Mexico; 8 Charité Universitätsmedizin Berlin, Berlin, Germany; 9 Andros Mannenkliniek, Arnhem, Netherlands; 10 Sunnybrook Health Sciences Centre, Toronto, Canada

**Keywords:** Prostate cancer, Cancer therapy

## Abstract

**Background:**

Androgen deprivation therapy (ADT) is foundational in the management of advanced prostate cancer (PCa) and has benefitted from a recent explosion in scientific advances. These include approval of new therapies that suppress testosterone (T) levels or inactivate its function, improvements in diagnostic and assay technologies, identification of lower therapeutic targets for T, discovery of the relevance of germline genetic mutations and identification of the benefits of sequential and combination therapies.

**Methods:**

This review discusses the clinical profiles of the most up-to-date options for ADT, best practices for managing patients with advanced PCa and future directions in therapy.

**Results and conclusions:**

Modern assay technologies reveal that bilateral orchiectomy results in a serum T level of approximately 15 ng/dL as compared to the historical definition of castration of T < 50 ng/dL. Evidence shows that lowering T levels to <20 ng/dL improves patient survival and delays disease progression. Routine monitoring of T in addition to prostate-specific antigen throughout treatment is important to ensure continuing efficacy of T suppression. New drugs that inhibit androgen signaling in combination with traditional ADT suppress T activity to near zero and have significantly improved patient survival. When personalizing ADT regimens physicians should consider a number of factors including initiation and duration of ADT, monitoring of T levels and PSA, the possibility of switching monotherapies if a patient does not achieve adequate T suppression, and consideration of intermittent vs. continuous ADT according to patients’ lifestyles, comorbidities, risk factors and tolerance to treatment.

## Introduction

After skin cancer, adenocarcinoma of the prostate is the most common type of cancer afflicting men in the United States, with more than 11.6% of males being diagnosed with prostate cancer (PCa) at some point during their lifetime [1–3]. From the first description and diagnosis dating back to the late eighteenth century, PCa is recognized as a hormone-dependent disease [4]. A clear target, the androgen receptor (AR) signaling pathway, has been identified as a primary objective for the development of effective therapies. In healthy males, the androgens testosterone (T) and its derivative dihydrotestosterone (DHT) are essential for cell survival and function of the prostate [5]. However, PCa cells exhibit excess activation of the androgen signaling pathway resulting in uncontrolled proliferation of tumor cells [6].

The initial discovery that hormones modulate prostate gland size and function, combined with the observation that PCa growth is influenced by androgen production, provided the basis for androgen deprivation therapy (ADT). ADT remains the foundational treatment of advanced PCa with its primary objective to reduce circulating levels of androgens [7]. The original form of ADT that remains in use worldwide is bilateral orchiectomy. While effective, this surgical procedure has been replaced with medical options as the gold standard where ADT drugs are available. There are still benefits to bilateral orchiectomy, such as cost savings compared to medical castration, which may be outweighed by concerns of psychological trauma to the patient and the irreversibility of the procedure.

The role of effective ADT has been further endorsed in recent years by the explosion of scientific advances confirming the importance of suppression of T activity in the management of advanced PCa. Such advances include the introduction of new hormonal therapies with novel mechanisms of action, improvements in diagnostic technologies, updates in science and data that have redefined optimal suppression targets for T, identification of the potential for sequencing and using combination therapies, increasing relevance of nadir T (the lowest level achieved), microsurges and escapes, all of which may impact the selection of therapies. With the emergence of new therapies that target androgen signaling through modes of action other than hormonal therapy (ADT), it would be more suitable to describe the class of drugs that result in the inactivation of the androgen signaling pathway as androgen-targeted therapy [8, 9].

## Evolving view of suppression targets for testosterone

Historically, the definition of castration has been suppression of T to a level lower than 50 ng/dL, based on radioimmunoassays developed in the 1960s that were less accurate when measuring lower levels of T. Advances in assay technology with greater sensitivities confirm that T levels following bilateral orchiectomy are approximately 15 ng/dL [7].

These findings, together with data that demonstrate improved survival and prolonged time to disease progression with lower levels of T, has led to consensus among PCa experts that a lower target, below 20 ng/dL, is desirable. Almost all PCa tumors will initially respond to ADT, although with long-term T suppression, some cell populations become refractory and elimination of T production from the testes is no longer sufficient to fully suppress tumor cell growth [10]. This is referred to as castration-resistant PCa (CRPC), which is determined by a rising PSA in an environment where T levels are castrate [11]. In CRPC, reactivation of AR pathways from multiple mechanisms occurs, including production of androgens by the adrenal glands and PCa cells themselves, androgen-independent activation of the AR, AR gene amplification or overexpression, constitutively active ligand-independent AR splice variants, and gain-of-function mutations involving the AR ligand-binding domain [12]. Despite this, continuation of T suppression to castrate levels remains important throughout the course of CRPC. One manifestation of the heterogeneity of tumor cell populations is that PCa cells exhibit varying degrees of androgen sensitivity [13]. Growth of androgen-sensitive cells will remain suppressed in a low T environment, hence the need for ongoing, effective ADT; however, growth of androgen insensitive cells will not be prevented. There is also a third compartment of partially resistant cells that only undergo apoptosis at very low levels of T.

A much less common form of PCa is small cell carcinoma that is highly malignant, presents with low PSA levels and has little dependency on AR signaling; patients with this tumor do not usually benefit from ADT [14]. Neuroendocrine differentiation can also occur in PCa that may lead to castration resistance before any rise in PSA [11].

There is increasing evidence that very low nadir T levels, particularly during the first few months of ADT, and absence of microsurges and escapes in T may be associated with improved clinical outcomes, including survival [14, 15]. This confirms the critical role of T in stimulation of PCa cells and emphasizes the importance of selecting an ADT with the greatest impact on T levels. During therapy, T should be monitored frequently to confirm achievement of targets, ideally to <20 ng/dL; if not, consideration should be given to improving patient compliance or selecting an alternative ADT.

## Expanding modes of action of hormonal treatments

**Fig. 1:**
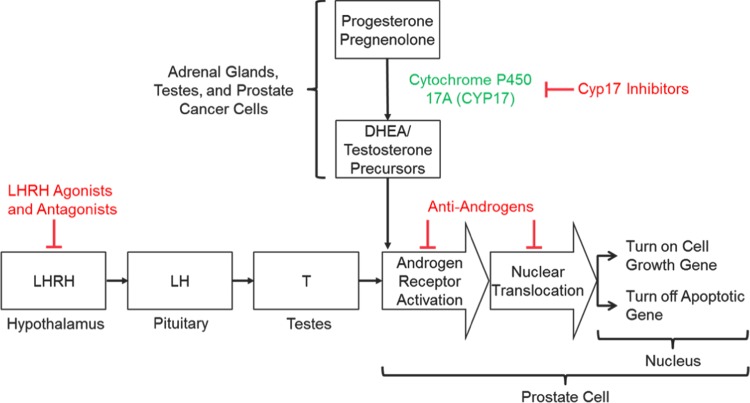
Mechanisms of androgen signaling inhibition

As PCa is a largely hormone-driven tumor, understanding the androgen signaling pathway, its role in cell growth, and identification of vulnerable points for manipulation is important when evaluating pharmacological treatments [16]. The centralized hormonal control of T production was the first element of the pathway to be investigated. T secretion is initiated in the hypothalamus with pulsatile release of LHRH, followed by binding to, and stimulation of, LHRH receptors in the anterior pituitary gland that cause the release of luteinizing (LH) and follicle-stimulating (FSH) hormones. LH stimulates receptors on Leydig cells in the testes to induce production of T. Suppression of this hypothalamic−pituitary−gonadal axis is the mechanism by which LHRH agonists (also referred to as gonadotropin-releasing hormone (GnRH)) and antagonists reduce circulating T levels [16] (Fig. 1).

Inhibition of enzymes involved in the biosynthesis of T is an additional therapeutic target. T is a steroid hormone that is derived from cholesterol through a cascade of biochemical reactions [17]. The final steps of biosynthesis involve the enzymes 17α-hydroxylase and C17,20-lyase that convert the precursors pregnenolone into the weaker androgen dehydroepiandrosterone, and progesterone into androstenedione. These enzymes are normally found in the testes and adrenal glands; however, some PCa cells can synthesize them and produce T outside of the control of the normal regulatory mechanisms. With effective inhibition of these enzymes, biosynthesis of T at all sites can be prevented (Fig. 1).

Additionally, the AR signaling pathway can be directly inhibited by antiandrogen molecules that prevent binding of androgen to the AR. In PCa cells, DHT binds to the AR in the cytoplasm, causing activated protein to translocate to the nucleus where it promotes the transcription of genes that regulate cell growth and survival. The AR binds directly to the promotor of genes at the AR element. Antiandrogens such as enzalutamide and apalutamide bind to the AR in the cytoplasm, disrupt the interaction between androgens and the AR thus preventing translocation of the AR into the nucleus and subsequent binding to DNA [18] (Fig. 1). Enzalutamide may also interact with coactivator function at the DNA binding site to prevent transcription [19].

Therefore, in patients with CRPC, there are multiple pharmacological routes through which the PCa cell can be deprived of androgenic stimulation, thereby preventing tumor cell growth and providing clinical benefit.

## Current armamentarium of drugs targeting reduction in testosterone production and action

### Antiandrogens

Some of the earliest drugs to be studied for treatment of PCa were antiandrogens, such as bicalutamide, flutamide, and nilutamide, nonsteroidal molecules available in oral dosage forms. A further antiandrogen, cyproterone acetate, is steroidal and has only been approved in Europe. As monotherapies, antiandrogens inhibit the binding of DHT to the AR but they do not reduce the level of serum T and are less effective than surgical castration or LHRH agonists in patients with metastatic PCa [20]. Antiandrogens are usually used concomitantly with LHRH agonists to reduce the clinical impact of the T surge related to the first dose, or dosed in combination with an LHRH agonist or antagonist to achieve “complete androgen blockade” (CAB) in men with metastatic PCa [21]. They are prescribed by some clinicians as monotherapy in patients with nonmetastatic disease who wish to preserve libido and avoid the metabolic effects of ADT [22].

### LHRH agonists and antagonists

LHRH agonists and antagonists were also among the first therapies developed to reduce androgen signaling in PCa. The pharmacological target is the LHRH receptor in the anterior pituitary gland. Continuous (i.e., nonpulsatile) serum levels of LHRH agonists stimulate the receptor and generate a transient surge in release of LH and T, followed by downregulation of the receptor over 2−3 weeks with reduction in LH and subsequent suppression of T production by the testes [23]. The amplitude of the surge depends on the baseline T; higher levels lead to greater surges [24]. Conversely, LHRH antagonists competitively and reversibly bind to and block LHRH receptors, inhibiting LH release and T suppression without an initial rise in T.

There are a number of LHRH agonist molecules with a range of drug delivery technologies to effect continuous, controlled release of drug. These include intramuscular (IM) leuprolide acetate (IM-LA), LUPRON^®^ with a microsphere technology [25] and ELIGARD^®^ subcutaneous (SC) leuprolide acetate (SC-LA) that utilizes the ATRIGEL^®^ Delivery System, a biodegradable, copolymer formulation [26]. SC-LA and IM-LA both have 1-, 3-, 4-, and 6-month formulations. Triptorelin pamoate (TRELSTAR^®^) also employs microspheres and is available in 1-, 3-, and 6-month IM formulations [27]. Goserelin acetate (ZOLADEX^®^) uses 1- and 3-month SC implants that require insertion under the supervision of a physician [28, 29]. Although not readily available, histrelin acetate (VANTAS^®^) is a 12-month SC implant inserted into the upper arm [30].

LA is by far the most common LHRH agonist used in the US, and 98−100% of patients receiving SC-LA and 93−100% of those receiving IM-LA achieved T levels below the US Food and Drug Administration (FDA) defined castration level of 50 ng/dL [25, 26, 31]. 95–99% of patients receiving triptorelin and 65−91% treated with goserelin achieved the same target. However the results are not directly comparable as the data are derived from different studies, albeit in similar patient populations. Limited head-to-head studies of LHRH agonists have not generally demonstrated differences in extent of T suppression; however, improved clinical outcomes have been identified in patients achieving T levels of <20 ng/dL and performance of each drug in achieving this target may be relevant when selecting an ADT agent.

In studies of SC-LA, 89−98% of patients achieved T < 20 ng/dL across all four doses [32]. Similar data for IM-LA are limited, although a retrospective, pooled analysis of the 4- and 6-month formulations found that 89−94% of patients achieved this level [33] and data from another study using the 1-month dose demonstrated 66% of patients reached T < 20 ng/dL [34]. For the 3-month formulation of triptorelin and the 1-month dose of goserelin, 25 and 55% of patients, respectively, achieved the target [35, 36]. Again, these data are from different studies so comparisons should be treated with caution.

Differences in pharmacokinetics (PK) between LHRH agonist therapies may also be relevant. In a phase 1 study of 1-month doses of IM-LA and SC-LA, serum LA levels in the SC-LA arm remained above the limit of quantitation (defined as effective) for 10−20 days longer than IM-LA. These PK differences resulted in very different PD profiles, with SC-LA suppressing LH and maintaining median T levels at castrate level for a longer period (at least 56 days vs. 35 days in SC-LA and IM-LA arms, respectively) [37]. These outcomes are likely attributed to differences in the controlled-release technologies and the data challenge the commonly accepted position that ADT therapies are interchangeable. Differences in duration or extent of T suppression reinforce the importance of routinely monitoring T levels to ensure target levels are achieved.

ADT also has important effects on prostate-specific antigen (PSA) levels. For SC-LA, they were reduced to normal (<4 ng/mL) in 91−95% of patients by study end [26] and for IM-LA, 63% of patients receiving the 3-month formulation and 51% (4-month dose) achieved this level at 12 and 32 weeks, respectively [25]. Goserelin demonstrated decreases of 93 and 94% in PSA levels for the 3.6 and 10.8 mg doses respectively, and reduction of 96% at the end of treatment for the 6-month formulation of triptorelin.

Data on nadir T are not readily available for these drugs. The association between nadir T during the first year of ADT and improved patient outcomes is a recent finding and the clinical trials of the LHRH agonists were conducted prior to recognition of its significance. However, the information available may provide additional evidence for determining overall effectiveness of the drugs and the data are presented in Table 1. In a pooled analysis of SC-LA pivotal trials, nadir T levels below 5, 10 and 20 ng/dL were achieved in 91, 97 and 99% of patients, respectively [38].

**Table 1:** Reported Lab Values for Available ADT Options

Generation	Drug (Reference)	Proportion of patients				
		T ≤ 50 ng/dL	T ≤ 20 ng/dL	Nadir T ≤ 10 ng/dL	No T Escape > 50 ng/dL	PSA decline >90% or to <4 ng/mL
1	Bicalutamide [121]					39
	Flutamide [122, 123]					13−40
	Nilutamide [124]					n/d
	Cyproterone acetate [125, 126]					4−70
2	SC-leuprolide acetate [26, 32, 38]	94−99	90−96	97	98−100	91−95
	IM-leuprolide acetate [25, 33, 127]	93−95	66−79	n/d	91−98	51−87
	Triptorelin [27, 31, 35, 128, 129]	93−98	25−79	n/d	93−99	81
	Goserelin [28, 36, 130]	65−91	55	n/d	91	n/d
	Degarelix [43, 45, 46, 89]	99−100	63	n/d	97−98	69−95
	Relugolix [64]	n/d	n/d	n/d	n/d	n/d
3	Abiraterone [131, 132]	n/d	n/d	n/d	n/d	19
	Enzalutamide [59, 60]					25−47
	Apalutamide [133]					42−43
	Darolutamide [134]					30

Gray cells: Not relevant as mechanism does not affect serum testosterone

Data should not be directly compared, as numerous sources, different doses, and time points are used

*n/d* no data

The safety profiles of the LHRH agonists are similar and they are generally well tolerated. The most common adverse effects (AE) are hot flashes, fatigue, sexual dysfunction, decreased erections, general pain, testicular atrophy, joint disorder, osteoporosis and metabolic alterations, consistent with the pharmacological action of T suppression. Additionally, increased risks of diabetes, cardiovascular events, and decreased bone density have been reported [39–41].

A single LHRH antagonist i.e., degarelix (FIRMAGON^®^) is approved for treatment of advanced PCa [42, 43]. Abarelix, the first drug in this class, was voluntarily withdrawn in May 2005 due to the occurrence of systemic anaphylactic reactions [44]. Degarelix is only available as a 1-month SC dose, requiring two initial injections (2 × 3 mL for 240 mg) followed by monthly doses of 4 mL (80 mg). LHRH antagonists competitively bind to the LHRH receptor, inhibit downstream LH signaling, and suppress T secretion. LHRH antagonism is not associated with an initial surge in T and suppression of T release is effective within 2−3 days.

Data on degarelix demonstrated 99−100% of patients achieved T < 50 ng/dL, although the data on reaching levels of <20 ng/dL were not reported [45]. In a group of eight patients with CRPC receiving an LHRH agonist where T levels were >20 ng/dL, a change to degarelix produced a decline to <20 ng/dL in five patients [46]. These data have not been confirmed in a randomized trial. Use of degarelix has been modest due to the lack of any dose exceeding 1 month and the frequency and severity of local injection-site reactions. However, due to the rapid fall in T and absence of surge, degarelix has been used to initiate ADT, with many patients then converted to a more convenient and better tolerated LHRH agonist for long-term treatment. Some patients that can tolerate degarelix continue to receive ongoing monthly doses [47]. Other AEs are related to T suppression and are similar to those seen with LHRH agonists, with the exception of a lower risk for cardiovascular (CV) events in patients with a history of CV disease and fewer musculoskeletal and urinary tract events [48, 49]. Degarelix appears to reduce FSH more than LHRH agonists (90 vs. 50%) although the mechanism of this difference is not clear. The clinical significance of this is controversial; however, there is some evidence that lower levels of FSH may be cardioprotective, particularly in men with preexisting CV disease, and may also produce less sarcopenia [45].

### Androgen pathway inhibitors

Antiandrogens and drugs that target the LHRH receptor represent first- and second-generation ADT options. Third-generation drugs have additional mechanisms of action and are collectively described as androgen pathway inhibitors (Table 2).

**Table 2:** Mechanisms of action for androgen-targeted therapy options

Therapeutic options	Mechanism of action
Orchiectomy	Surgically remove both testes to reduce T production
Antiandrogens	Block the androgen receptor to reduce effects of T signaling in the cell
LHRH agonist	Overstimulate the pituitary gland to downregulate the GnRH receptor and decrease LH production, which lowers T production in the testes
LHRH antagonists	Block the GnRH receptor to decrease LH production, which lowers T production in the testes
Androgen pathway inhibitors	Target the androgen pathway to inhibit T synthesis or reduce AR signaling

Abiraterone acetate (ZYTIGA^®^) is an oral, androgen biosynthesis inhibitor that blocks T production through inhibition of the enzyme CYP17 [50]. It is administered in combination with prednisone and with ongoing ADT, and is effective in reducing androgen production from all sources including the testes, adrenal glands, and PCa cells. Several trials have found that abiraterone in combination with ADT profoundly suppresses T to lower levels than are generally seen with an LHRH agonist alone [51].

Abiraterone was first studied in patients with metastatic CRPC (mCRPC) and disease progression after docetaxel; it lengthened radiographic progression-free survival (rPFS) by 2 months and increased overall survival (OS) by 3.9 months [52]. In docetaxel-naïve patients, rPFS increased by 8.3 months and OS by 4.4 months [53]. More recently, the LATITUDE trial studied abiraterone in men with high-risk, metastatic, castration-sensitive PCa, leading to recent approval by FDA in this indication [54]. Data showed that abiraterone increased OS (HR 0.62; 95% CI 0.51–0.76; *P* *<* 0.0001) and improved many secondary clinical endpoints [54]. The STAMPEDE trial investigated abiraterone in a similar group of patients where 20% were node-positive, 27% had high-risk, locally advanced disease, and 5% experienced biochemical failure [55]. Radiotherapy was mandatory for patients with high-risk, locally advanced disease and optional for patients with node-positive disease. The results confirmed that abiraterone significantly decreased the number of deaths (HR 0.63; 95% CI 0.52–0.76; *P* *<* 0.001). These data demonstrate that adding abiraterone to ADT and inducing very low levels of T through two distinct and complimentary pathways has the potential to further improve outcomes [55–57]. This implies that T continues to stimulate growth of PCa cells, even when levels are below 20 ng/dL. Therefore, suppression of T to near zero can bring additional positive benefits to patients with advanced PCa.

In addition to the expected AEs associated with T suppression, abiraterone may also produce events associated with mineralocorticoid toxicity (i.e., hypertension, hypokalemia, and fluid retention) and liver function abnormalities, some of which may be severe and include fulminant hepatitis and acute liver failure. As a result, serum transaminases and bilirubin levels should be assessed prior to initiating treatment, every 2 weeks for the next 3 months and monthly thereafter. Blood pressure and potassium levels should be measured monthly. Due to the concomitant administration of prednisone, additional AEs are possible such as confusion, excitement, restlessness, headache, nausea, and vomiting [58].

Enzalutamide (XTANDI^®^) is an oral, nonsteroidal antiandrogen indicated for the treatment of patients with mCRPC. It competitively binds to the AR at the androgen-binding site and also inhibits nuclear translocation and interaction of the AR with DNA. This prevention of AR-dependent transcription causes decreased cell proliferation and induces cell death. Enzalutamide blocks the action of T at the cellular level regardless of where it is derived and is administered in conjunction with continuing ADT. In a study of enzalutamide vs. placebo in patients with mCRPC who had received prior chemotherapy, median OS was improved (18.4 vs. 13.6 months, respectively, *P* *<* 0.0001) [59]. In patients naïve to chemotherapy, the estimated median OS for enzalutamide was not reached (82% of patients remained alive at 18 months) compared to median OS of 31.0 months in the placebo group (hazard ratio, 0.73; 95% CI 0.63−0.85; *P* *<* 0.001) [60]. Hence, improved outcomes similar to those for abiraterone are seen for enzalutamide, but they are achieved via a completely different mechanism. Intriguingly, this raises the question of whether ADT plus the combination of these two new drugs may result in even greater benefits, and studies investigating this are underway [51, 54, 55]. Regarding safety of enzalutamide, in addition to the common expected AEs for an AR inhibitor, seizures and posterior reversible encephalopathy syndrome have been seen on rare occasions, likely due to the drug crossing the blood−brain barrier [61].

The recent FDA approval of apalutamide (ERLEADA^TM^), an oral, nonsteroidal antiandrogen that blocks the action of T by binding to the ligand-binding domain of the receptor for the treatment of nonmetastatic CRPC, further confirms the benefit of androgen pathway inhibition across the disease continuum [62]. Apalutamide was designed to supersede the current androgen pathway inhibitors by overcoming AR-related resistance mechanisms. Patients with nonmetastatic CRPC received apalutamide or placebo in combination with ADT or bilateral orchiectomy. Data demonstrated metastasis-free survival of 40.5 months for the apalutamide group vs. 16.2 months for placebo. The most common adverse reactions were fatigue, hypertension, rash, and diarrhea [62]. The improvements in survival demonstrated by apalutamide and enzalutamide, which block the action of T^61,62^ and abiraterone, which blocks T synthesis [50] across a wide spectrum of advanced disease implies that inhibition of T signaling may be of central importance in delaying or suppressing metastases.

### Drugs in development targeting the androgen pathway

Other ADT drugs include darolutamide (ODM-201) and relugolix that are not yet approved. Darolutamide is an oral, nonsteroidal antiandrogen with a similar mode of action to enzalutamide and apalutamide. In a 12-week phase 2 study, darolutamide demonstrated a PSA response rate of 29% in the low, 33% in the mid, and 33% in the highest dose group [63].

Relugolix is an oral GnRH antagonist in phase 3 development. In healthy males the drug was readily absorbed and reduced mean serum T levels within 6 h of dosing; however, a food effect reduced exposure by 50%. T recovered rapidly following cessation of treatment [64].

Patients may prefer oral dosing over injections due to the convenience of not requiring a clinic visit for injections and the avoidance of injection-site AEs; however, there may be disadvantages. Compliance with oral dosing is rarely 100%, especially for long-term treatments where dosing may be required for months or years and particularly where the underlying illness is asymptomatic. Missed doses may compromise efficacy, which may be critical when the illness is serious or life threatening e.g., use of statins or antihypertensives in patients with cardiovascular disease and dosing of cancer treatments [65]. With ADT, this issue can be avoided and 100% compliance achieved if the therapy is given on schedule via long-acting injection. Due to the high daily doses of drug required for the androgen pathway inhibitors, depot injections may not be feasible.

## Selection of ADT regimen

Due to the recent advances in treatment for advanced PCa, some patients may live for many years with their disease. Physicians need to assess the most appropriate drug and dosing regimen for each patient and make adjustments to ensure targets are achieved and maintained. The AE profiles for all ADT drugs are similar due to the impact of T inhibition and they are generally well tolerated, except maybe with respect to injection-site reactions and specific rarer issues such as central nervous system effects and liver function test abnormalities. Therefore efficacy, including achievement of targets, may be the most important element to consider when selecting a regimen.

### Initiation and duration of ADT

ADT is typically the first systemic treatment used after local therapy options have been exhausted or deemed insufficient, although it is also used earlier as an adjunct to surgery or radiation. Once initiated, ADT is generally continued throughout the course of PCa treatment, including during CRPC when androgen pathway inhibitors, sipuleucel-T or other drugs are introduced.

### Dosing interval

LHRH agonists and antagonists offer extended release formulations that range from 1 to 6 months in duration. A 12-month implant of histrelin is available, but not widely used. Some patients may initially receive a 3- or 6-month dose of an LHRH agonist, whereas others may transition from an initial 1-month dose to a longer duration option. Selection of the appropriate dose interval should be a shared decision between the physician and patient based on preference and appropriate disease management.

### Switching monotherapies

Most patients receive an LHRH agonist at the initiation of ADT, although some physicians use degarelix to obtain rapid suppression of T without a surge, then switch to an LHRH agonist [42, 45]. In addition to changing drugs for scheduling convenience, an alternative drug or formulation should be considered when T control is inadequate. Regular assessment of T will determine success of ADT, and high levels may be due to incorrect preparation and administration or failure of the drug itself. Errors at the time of injection may cause irregularities in the release of drug and consequent lack of efficacy—additional training of staff should correct this. If inadequate effect of the drug is the cause, a switch to a different drug or release technology should be considered [66]. Once switched, T levels should be monitored to ensure they are maintained at <20 ng/dL [67].

### Intermittent vs. continuous ADT

Intermittent dosing, referred to as intermittent androgen deprivation (IAD), may be offered to some patients. In murine mammary carcinoma, IAD delayed tumor progression and this result formed the basis of using IAD in patients [68]. Patients on IAD may start their off-treatment period when PSA is <4 ng/mL and will have PSA routinely monitored and likely resume ADT when it rises to 10 ng/mL. IAD has the potential to reduce AEs associated with ADT as T levels recover, and also lower costs. IAD may be an option for some patients with nonmetastatic PCa and a modest risk of progression who experience significant ADT-related AEs, if they had a good initial response to ADT (PSA < 0.2 ng/mL) [67]. It may also be an option for patients who have a low burden of metastases and a complete biochemical response to induction of ADT therapy [13]. A trial of discontinuation of ADT is unlikely to have serious adverse consequences and some patients will experience a prolonged off treatment interval. However, if PSA levels rise rapidly, then continuous ADT should be started and maintained. Importantly, the SWOG trial failed to demonstrate that IAD was noninferior to continuous ADT in patients with metastases [69]. Most trials of IAD have shown some improvements in quality of life (QoL) but only small reductions in AEs during the off-treatment phase [70]. ICELAND, the most recent IAD trial, did not show significant differences between IAD and continuous ADT in health-related QoL or AEs [71]. Furthermore, recent analysis of a large trial of IAD in patients with metastases suggested an increase in CV events in patients who received the intermittent regimen [72].

Based on the evidence, patients and physicians should discuss the risks and benefits of IAD and agree on whether it is a safe and effective option.

## Laboratory evaluations in the management of prostate cancer

PCa is an almost unique therapeutic area in that regulatory approvals of drugs such as LHRH agonists and antagonists are based on achievement of endpoints for a defined biochemical surrogate (castration levels of T) as opposed to clinical outcomes. A similar example would be the approval of statins based on reductions in LDL cholesterol before CV clinical endpoints had been achieved. This concept supports the use of laboratory measurements during PCa treatment as being appropriate to assess response to therapy, tumor microenvironment, state of disease progression and prognosis. Serum PSA levels are routinely evaluated as a biomarker of PCa diagnosis and progression [73]. However, T may also be associated with clinical significance, including nadir levels, microsurges, and escapes during ADT; additionally, measurement of FSH may also be relevant [14, 15, 74–77].

### Testosterone suppression target of less than 20 ng/dL

Setting goals for successful suppression of T during ADT for PCa should be based on evidence, measurement technologies, and relevance to patient outcomes. Although all forms of ADT aim to suppress T to castration levels, there has been disparity regarding the target. Recent advancements in assay technologies have enabled quantification of T levels down to extremely low levels (e.g., 2 ng/dL) [78, 79] and helped establish that T levels in surgically castrated men are substantially lower than originally reported [7, 78]. Based on these results, the European Association of Urology (EAU) updated its PCa guidelines in 2014 to define the target for T during ADT as <20 ng/dL [80]. Despite this, and a similar recommendation for a 20 ng/dL threshold from the Bethesda consensus (a review conducted by US urologic oncologists) [81], the National Comprehensive Cancer Network and American Urological Association have not yet changed their recommendations. Furthermore, the FDA has not amended its regulatory target of >90% of patients achieving and maintaining T < 50 ng/dL [82] for new drug approvals. Studies have also found that patients with T levels below 20−32 ng/dL benefited from a delay to CRPC and significantly lower risk of death compared to those with higher T levels [14, 83]. Given the weight of this evidence, it may now be appropriate to require ADT drugs to achieve and maintain T levels of <20 ng/dL, and for all clinical treatment guidelines to reflect this lower target [14, 15, 21, 81, 84, 85].

### Nadir testosterone

Recent evidence suggests that nadir T during ADT correlates with a delay in progression to CRPC [15]. A significant improvement in cancer-specific survival and increased time to androgen-independent progression among patients with nadir T of <20 ng/dL compared those with T > 20 ng/dL has been demonstrated, and patients with nadir T of ≥50 ng/dL had a greater risk of death from PCa compared to those with lower levels. Klotz et al. also found that nadir T during the first year of continuous ADT correlated with increased time to androgen-independent progression and cancer-specific survival [15]. Furthermore, a significant difference in time to castrate resistance was found between patients who reached a nadir T of <20 ng compared to 20−50 ng/dL and >50 ng/dL. A multivariate analysis by Kamada et al. showed that nadir T below 20 ng/dL was a significant prognostic factor for OS [84]. A model for explaining prolonged survival in patients who achieve nadir T below 20 ng/dL was described by Klotz; it characterizes hormone-naïve PCa as having three distinct cell subpopulations: androgen insensitive stem cells that have functionally deficient or absent AR, partially androgen sensitive, and androgen sensitive cells. In the absence of androgen, as with a very low nadir T, surviving cells are the fully androgen insensitive stem cells. These cells repopulate with an AR-expressing, androgen-sensitive phenotype. When nadir T is not sufficiently low, partially androgen insensitive cells persist with accelerated progression to androgen resistance. Although this suggests that nadir T is an important metric for PCa prognosis during the first year of ADT, additional data are needed to assess clinical benefits with subsequent years of therapy.

Data on nadir T levels are not generally available for hormonal therapies; however, a study for SC-LA demonstrated that nadir T levels below 5, 10 and 20 ng/dL were achieved in 91, 97 and 99% of patients, respectively [38]. Additional evidence will be required before the clinical implications of this can be fully understood.

### Testosterone surges, escapes, and microsurges

Following the first injection of LHRH agonists, a surge in T due to hyperstimulation of the GNRH receptor will occur, followed by downregulation and subsequent inhibition of production of T by the testes. Escapes in T (often defined as a level ≥50 ng/dL) are possible where T levels rise before a subsequent dose. Morote et al. found that T levels above 32 ng/dL resulted in a mean PFS of 88 months compared to 137 months for patients who did not experience escapes (*P* *<* 0.03). Studies have also shown improved survival free of androgen-independent progression when T escapes are minimized [85].

Microsurges in T may occur following a subsequent dose if suppression of the hypothalamus−pituitary−gonadal axis has not been effectively maintained; in some cases, this can be due to a delay in administration of the next injection [47, 86]. The definition of a microsurge is not standardized; it is sometimes defined as an absolute increase in T of 25 ng/dL [87]. Additionally, the clinical implications of microsurges remain to be identified [87].

While the clinical significance of surges in T with the first dose of an LHRH agonist is unknown for most patients (apart from clinical flare in patients with urinary obstruction or spinal cord compression), it may be desirable to avoid the consequences of this initial rise and this can be achieved by coadministration of an antiandrogen, or initiation of ADT with an LHRH antagonist.

### Follicle-stimulating hormone and cardiovascular risk

The function of FSH in healthy males is to upregulate androgen-binding proteins to maintain normal sperm production and to stimulate sperm growth. FSH is released by the pituitary in response to GnRH and has been implicated as a potential factor in the development of atherosclerosis during ADT. A higher serum FSH correlates with formation of lipid droplets and upregulated genes encoding for lipogenesis proteins. There may be differences in the FSH profile between ADT drugs, and FSH microsurges may occur in parallel with T microsurges [88–90]. It has been observed from comparative studies with abarelix that LHRH agonists cause a surge in serum FSH on day 2 followed by a decline, whereas the antagonist led to a rapid and sustained decrease [91]. Klotz et al. also observed that degarelix rapidly decreased FSH to levels <90% of normal whereas IM-LA produced an initial increase in FSH levels followed by a decrease to approximately 50% of normal [45]. Data are not available for FSH changes with SC-LA or other LHRH agonists. Interestingly, surgical orchiectomy results in very high FSH levels due to the loss of inhibin secretion by Sertoli cells. A retrospective chart review of the Taiwan National Health Insurance Research Database found that while no significant difference in CV risk was detected during the median follow-up time of the study (3.3 years) between patients who received orchiectomy vs. LHRH agonist therapy, during the short-term follow-up (first 1.5 years) there was an association with higher risk of CV ischemic events in the orchiectomy group (HR, 1.40; 95% CI 1.04−1.88) [92].

Since older men are at increased risk of CV disease, it is relevant to evaluate if specific drugs to treat PCa impact this risk. Emerging data implicate a role for FSH in promoting the development of factors associated with CV disease in PCa patients [93]. While the findings require confirmation through further research, it may be of value to assess FSH levels during ADT, particularly if the patient is at high risk for CV disease, to monitor AEs and adjust treatment accordingly [93, 94]. It is critical to identify and manage CV risk factors in all PCa patients, especially with respect to treating hypertension, hyperlipidemia, diabetes, etc.

The evidence from prospective, randomized trials of LHRH drugs, which typically exclude patients with significant CVD suggests there are no significant differences in CVD risk when using LHRH agonists or antagonists; this may be a “healthy cohort” effect [95, 96]. In contrast, in patients with prior CV events, LHRH agonists may be associated with longer term CV risk. A retrospective analysis of pooled data from six randomized trials comparing degarelix to LHRH agonists found that among men with preexisting CV disease, the risk of cardiac events within 1 year of initiating therapy was significantly lower in those treated with the LHRH antagonist compared with the LHRH agonists [49]. However, limitations were that it was a secondary analysis and CV events were reported as AEs rather than independent study endpoints. Furthermore, in a prospective, randomized trial of radiation compared to a combination of radiation and ADT in men with localized, high risk PCa, no significant increase in CV risk with ADT was found [97]. The association between FSH levels and the risk for CV events, and the differential effect of ADT on this risk warrants further study. The PRONOUNCE trial comparing degarelix and IM-LA is designed to investigate differences between these two drugs in patients with CV risk [98].

Beyond the debate on ADT-related CV morbidity, possibly the most important behaviors that patients can embark on to improve their CV health are to eat a healthy diet, exercise regularly, lose weight and see a physician to manage their medical CV risk factors including effective treatment of hypertension, hyperlipidemia and diabetes. Being successful in changing these lifestyle factors and improved medical management are likely to have a greater positive impact on their CV health than selection of a different ADT option.

### Prostate-specific antigen

PSA is a protease produced by the epithelial cells of the prostate gland that liquefies semen; it is secreted into both semen and blood [99]. PSA does not affect PCa cell proliferation, but is a marker of it due to its correlation with AR activity [100]. The promoter of the PSA gene includes several binding sites for the AR and activation of the AR by T binding leads to transcription and translation of PSA [101]. Prolonged PSA doubling time is associated with improved OS in patients with CRPC; a median PSA doubling time <45 days correlated with 16.5 months median survival compared to 26.4 months for patients with a PSA doubling time of ≥45 days [102, 103].

As a biomarker of PCa progression, PSA is relied upon by physicians as a determinant of initiation of more advanced treatments. However, due to limitations of PSA in detecting transient fluctuations in AR signaling [104, 105], evaluation of serum T provides a more timely and accurate assessment of the effects of ADT and identification of T escapes.

## Recommendation for routine testosterone testing

T levels should be measured regularly in men receiving ADT to ensure T suppression is being maintained to target; this does not appear to be the case in routine clinical practice. EAU guidelines recommend that T testing is performed 3 months after the first dose of ADT and repeated every 3−6 months thereafter. ADT use is often assumed to be a proxy for adequate T suppression to castrate levels; however, neglecting to assess T will fail to identify levels above target, microsurges, and escapes. Adding a T test to the regular PSA assessment is simple to implement and would address these concerns.

With a rise in PSA and progression to CRPC, T testing and management remain important. Confirmation that T is castrate at time of CRPC diagnosis is critical, and continuance of regular testing should confirm effective T suppression and prevention of repopulation of partially androgen-sensitive tumor cells. An incorrect diagnosis of CRPC may prompt the use of additional, more costly, and possibly more toxic therapies in patients who do not yet require them [106].

The most widely used assay for T determinations is the chemiluminescent immunoassay which is reliable at the higher levels of T typically measured in men being evaluated for androgen deficiency or infertility (i.e., >50 ng/dL), but may not be accurate at the very low levels seen in men on ADT (<20 ng/dL) [107]. Liquid chromatography-tandem mass spectroscopy (LC-MS/MS) is the most sensitive test available for detecting low levels of T [108], although it is more costly and labor intensive. Clinicians should be encouraged to communicate with their laboratory colleagues to ensure that T assays conducted while monitoring patients undergoing ADT have an appropriate lower limit of quantification, ideally LC-MS/MS or an immunoassay that has been validated to detect low levels of T. Additionally, consistent use of the same laboratory and the same assay for all T measurements from a patient would provide the highest level of comparability of patient lab values over the course of treatment.

## Future directions

A main focus for ongoing research is to establish whether the clinical benefits seen with abiraterone and enzalutamide in patients with mCRPC can be extended to all stages of the disease and to other androgen pathway inhibitors. Recent data with abiraterone (high-risk, metastatic castrate-sensitive PCa), apalutamide (nonmetastatic CRPC) and enzalutamide (mCRPC) seem to confirm the importance of inhibiting the androgen signaling pathway throughout the entire course of advanced disease. Additional trials studying abiraterone, enzalutamide, darolutamide, and apalutamide are currently recruiting patients and studies of combinations of androgen-targeted therapy drugs with different mechanisms are also underway [109–117].

Personalization of PCa therapy beyond identification of patients that benefit from ADT is achievable in the twenty-first century. As more therapeutic options become available and more clinically relevant tumor/genetic markers are identified, outcomes will improve by personalization of treatment strategies. Prospective trials can be undertaken to confirm that achieving and maintaining T levels <20 ng/dL result in improved clinical outcomes. Promising areas of research into genetic testing that identify levels of disease risk include the discovery of a higher incidence of germline mutations in DNA-repair genes, such as *BRCA2*, *ATM*, *CHEK2*, *BRCA1*, *RAD51D*, and *PALB2* in men with metastatic PCa compared to those with localized disease [118]. Future research will fully characterize the clinical significance of these gene mutations and determine how selection of therapies may be influenced and personalized by genotype. Recent studies have found that patients with mCPRC who have failed several lines of treatment and tested positive for germline or somatic DNA repair mutations show some response to PARP inhibitors [119]. Similarly, some patients with mismatched mutations experience dramatic responses to PDL-1 inhibitors [120]. Beyond these two examples, there is huge potential for finding benefits of new combinations of drugs or expansion in the indications of drugs. The development of tailored treatments based on patient-specific factors will ensure patients benefit from these new scientific advances.

## Conclusions

The management of advanced PCa has undergone a revolution over the last decade with the emergence of new science and data in androgen-targeted therapies. Patients are living longer and benefit from improved outcomes with the widespread use of new drugs such as abiraterone, enzalutamide, and apalutamide. These drugs, in combination with ADT, dramatically inhibit the availability of T to the tumor by near complete inhibition of the androgen signaling pathway. Additional studies on the benefit of these and other androgen pathway inhibitors in all stages of advanced PCa will likely produce similar results and confirm the importance of suppression of T to <20 ng/dL. Monitoring of T is essential to ensure success in achievement of this target.

LHRH agonists are the most widely used form of ADT due to their ability to provide long-lasting T suppression from single, well-tolerated injections lasting up to 6 months. As monotherapy, very low levels of T including nadirs less than 5 mg/dL are achieved by some drugs.

New products in development are employing novel mechanisms with greater potency or selectivity, or enhanced delivery to further improve on current therapies. Additionally, improvements in genetic testing deliver the potential for personalization of therapies to optimize efficacy and safety.

These exciting scientific advances in the management of advanced PCa using androgen-targeted therapies bode well for further improving the lives of the millions of patients living with this disease.
